# Can a quality improvement intervention improve person-centred maternity care in Kenya?

**DOI:** 10.1080/26410397.2023.2175448

**Published:** 2023-03-01

**Authors:** May Sudhinaraset, Katie M. Giessler, Michelle Kao Nakphong, Meghan M. Munson, Ginger M. Golub, Nadia G. Diamond-Smith, James Opot, Cathy E. Green

**Affiliations:** a Associate Professor, Community Health Sciences, Los Angeles (UCLA), University of California, Los Angeles, CA, USA.; b Senior Research Analyst, Institute for Global Health Sciences, San Francisco (UCSF), University of California, San Francisco, CA, USA; c Doctoral Student, Community Health Sciences, Los Angeles (UCLA), University of California, Los Angeles, CA, USA; d Program Manager, Jacaranda Health, Nairobi, Kenya; e Senior Research and Business Development Manager, Innovations for Poverty Action, Nairobi, Kenya; f Assistant Professor, Epidemiology and Biostatistics, University of California, San Francisco, CA, USA; g Senior Research Associate, Innovations for Poverty Action, Nairobi, Kenya; h Senior Improvement Advisor, Jacaranda Health, Nairobi, Kenya

**Keywords:** Maternal health, women’s experiences of care, person-centred maternity care, maternity care, quality of care, respectful maternity care, quality improvement, intervention, Quality Improvement Collaborative, Kenya

## Abstract

Few evidence-based interventions exist to improve person-centred maternity care in low-resource settings. This study aimed to understand whether a quality improvement (QI) intervention could improve person-centred maternity care (PCMC) experiences for women delivering in public health facilities in Kenya. A pre–post design was used to examine changes in PCMC scores across three intervention and matched control facilities at baseline (*n* = 491) and endline (*n* = 677). A QI intervention, using the Model for Improvement, was implemented in three public health facilities in Nairobi and Kiambu Counties in Kenya. Difference-in-difference analyses using models that included main effects of both treatment group and survey round was conducted to understand the impact of the intervention on PCMC scores. Findings suggest that intervention facilities’ average total PCMC score decreased by 5.3 points post-intervention compared to baseline (95% CI: −8.8, −1.9) and relative to control facilities, holding socio-demographic and facility variables constant. Additionally, the intervention was significantly associated with a 1.8-point decrease in clinical quality index pre–post-intervention (95% CI: −2.9, −0.7), decreased odds of provider visits, and less likelihood to plan to use postpartum family planning. While improving the quality of women’s experiences during childbirth is a critical component to ensure comprehensive, high-quality maternity care experiences and outcomes, further research is required to understand which intervention methods may be most appropriate to improve PCMC in resource-constrained settings.

## Résumé

Rares sont les interventions à base factuelle qui cherchent à améliorer les soins de maternité centrés sur la personne dans les environnements à faibles ressources. Cette étude visait à comprendre si une intervention d’amélioration de la qualité pouvait garantir une meilleure expérience des soins de maternité centrés sur la personne (SMCP) à des femmes accouchant dans des établissements de santé publique au Kenya. Elle a utilisé un schéma avant/après pour évaluer les changements dans les scores des SMCP pour trois établissements d’intervention et témoins correspondant initialement (n = 491) et postérieurement (n = 677). Une intervention d’amélioration de la qualité, utilisant le modèle d’amélioration, a été mise en œuvre dans trois établissements de santé publique dans les comtés de Nairobi et Kiambu au Kenya. Des analyses avec la méthode des doubles différences utilisant des modèles qui incluaient les principaux effets aussi bien du groupe de traitement que de la série d’enquête ont été menées pour comprendre l’impact de l’intervention sur les scores des SMCP. Les résultats donnent à penser que le score total moyen des SMCP dans les établissements de l’intervention a diminué de 5,3 points après l’intervention, par comparaison avec la période initiale (IC 95%: −8,8, −1,9) et en rapport avec les établissements témoins, en conservant les variables sociodémographiques et des établissements constantes. De plus, l’intervention était associée de manière significative à une diminution de 1,8 point de l’indice de qualité clinique avant-après l’intervention (IC 95%: −2,9, −0,7), une probabilité décroissante de visites des prestataires et une moindre vraisemblance de la volonté d’utiliser une planification familiale du postpartum. Si l’amélioration de la qualité de l’expérience des femmes pendant l’accouchement est un élément capital pour assurer des expériences et des résultats complets et de qualité des soins de maternité, des recherches complémentaires sont nécessaires pour comprendre quelles méthodes d’intervention sont les plus adaptées pour améliorer les SMCP dans les environnements aux ressources limitées.

## Resumen

Existen pocas intervenciones basadas en evidencia para mejorar la atención materna centrada en la persona en entornos con escasos recursos. El objetivo de este estudio era entender si una intervención para el mejoramiento de la calidad (MC) podía mejorar las experiencias de atención materna centrada en la persona (AMCP) de mujeres que dan a luz en unidades de salud pública en Kenia. Se utilizó un diseño pre–post para examinar los cambios en puntajes de AMCP en tres unidades de intervención y de control emparejadas en la línea de base (n = 491) y en la línea final (n = 677). Se ejecutó la intervención de MC, utilizando el Modelo de Mejoramiento, en tres unidades de salud pública en los condados de Nairobi y Kiambu, en Kenia. Se realizaron análisis de diferencias en diferencias utilizando modelos que incluían los principales efectos del grupo de tratamiento y la ronda de la encuesta, con el fin de entender el impacto de la intervención en los puntajes de AMCP. Los hallazgos indican que en las unidades donde se realizó la intervención, el puntaje total promedio de AMCP disminuyó en 5.3 puntos post-intervención comparado con la línea de base (IC al 95%: −8.8, −1.9) y relativo a las unidades de control, manteniendo constantes las variables sociodemográficas y de las unidades de salud. Además, la intervención se asoció de manera significativa con una disminución de 1.8 puntos en el índice de calidad clínica pre–post intervención (IC al 95%: −2.9, −0.7), menores probabilidades de visitas de prestadores de servicios, y menor probabilidad de utilizar planificación familiar posparto. Aunque mejorar la calidad de las experiencias de las mujeres durante el parto es un componente fundamental para garantizar experiencias y resultados de atención materna integral de alta calidad, se necesitan más investigaciones para entender qué métodos de intervención son los más indicados para mejorar la AMCP en entornos con recursos limitados.

## Introduction

Preventable maternal mortality is a major concern globally. Almost all deaths (99%) occur in developing countries, with half in sub-Saharan Africa.^1^ Kenya has seen improvements in the past two decades, though maternal deaths remain high, with a maternal mortality ratio of 342 deaths per 100,000 live births.^2^ Consequently, the Government of Kenya has made great efforts to curb maternal mortality in the country, including introducing a free maternal healthcare policy in 2013.^3^ While this has led to increases in deliveries in healthcare facilities, there have been few significant changes in maternal or neonatal mortality rates,^3^ pointing to poor quality of care as a driving factor.

The World Health Organization prioritises person-centred maternity care (PCMC) as an important component of quality of care.^4^ PCMC places the woman and her family at the centre of care, involving her in decisions and respecting and responding to her needs, values, and preferences.^5^ Improving PCMC may facilitate improved experiences for pregnant women; encouraging them to return to the facility for general and reproductive healthcare, as well as subsequent births.^6^ PCMC may improve postnatal care and lower new-born complications,^7^ thus potentially decreasing morbidity and mortality among women and babies. A recent literature review of PCMC interventions found mixed results in improving person-centred care outcomes, and there was no clear relationship between interventions and clinical quality of care.^8^ Examining promising interventions to improve PCMC is needed.

Quality Improvement (QI) interventions are one approach to improve processes and outcomes in healthcare settings. Specifically, Quality Improvement Collaboratives (QICs) bring together multidisciplinary teams from different organisations to work systematically to improve common healthcare challenges.^9^ Mixed results have been reported regarding the efficacy of QICs in low- and middle-income countries (LMICs).^10,11^ One QIC study in Ghana reported notable decreases in infant and under-5 mortality,^12^ while another conducted in Nigeria found no differences for retention in care at 6 months postpartum in an HIV prevention programme.^13^ Despite mixed evidence for success, QICs are popular for their potential to create long-term change, engage actors across various settings, and build broader communication platforms compared to other QI strategies.^14^ To our knowledge, no other studies have used a QIC approach to improve PCMC. The aim of this study was to assess the effectiveness of a facility-based QIC in improving patient experiences of delivery care in three hospitals in Kiambu and Nairobi counties. Additionally, the study assessed the indirect impact of the intervention on other health outcomes including clinical quality, provider visits, overall quality rating and satisfaction, experiences of delivery problems, and plans to use a family planning method.

## Materials and methods

### Study intervention, study design, and setting

**Figure 1.**
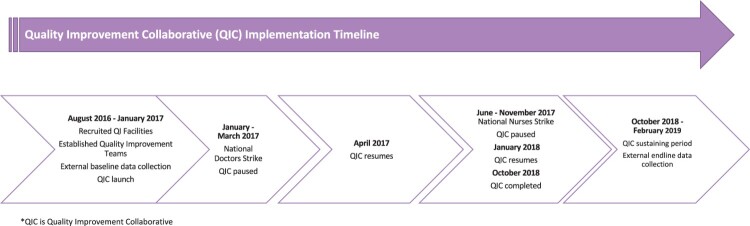
Timeline and activities for the quality improvement collaborative.

Three government-run health facilities in Kiambu and Nairobi counties in Kenya were recruited to join a QIC to improve aspects of person-centred care in maternity and family planning services in their facilities. Implementation of the QIC was led by Jacaranda Health, a Kenyan non-profit organisation that works with the National Ministry of Health and county governments to improve quality of care in health facilities. See Figure 1 for an overview of the timeline and activities for the QIC. In 2016, the study team convened a meeting in Nairobi, Kenya, including Kenyan researchers and implementers from Jacaranda Health and KEMRI, to design the protocol for the quality improvement implementation and evaluation. Site selection was determined by the facility’s delivery volume (i.e. at least 50 deliveries a month on average), location (i.e. Kiambu and Nairobi counties), provision of family planning and maternity care, and recommendations from their respective county governments (e.g. support of local county officials). Those with lower delivery volumes, outside of the counties of interest, those without provision of family planning or maternity care services, and without support of local county officials were not included. Intervention facilities were matched with similar control facilities based on urbanicity, patient volume, and staffing. None of the six facilities had previously participated in a QI intervention that was focused on improving PCMC.

Each of the three intervention facilities was asked to establish a QI team consisting of nurses and midwives from the maternity and family planning departments and non-clinical support staff (e.g. data clerks). External QI experts engaged facility leadership at the outset to understand the assets and limitations within the facility that would impact the effectiveness of the QI work, such as availability of personnel and supplies, and they worked to generate buy-in from leadership to support the project activities. Prior to the rollout of implementation, intervention facility leadership was engaged to lay out expectations, timelines, and procedures related to their involvement. Jacaranda Health led the engagement of health facility leadership, working closely with facility leadership to identify potential participants for the QIC, understand existing resources through a needs assessment, and gained buy-in from healthcare management and providers. Potential PCMC improvement topics were identified from a scale developed to measure PCMC in this setting (see measures section for more details),^15^ and were selected for the QIC based on poor performance at baseline for the topic across all three intervention sites. QI teams used the Model for Improvement (MFI) to set aims for improvement, establish common measures to capture improvement data, identify change strategies that appeared likely to improve PCMC behaviours, and test these using Plan-Do-Study-Act (PDSA) cycles within their respective facilities.^16^ QI teams conducted exit interviews of patients continuously throughout the study to assess whether change strategies led to improvement in PCMC experience. Teams met weekly with external QI experts from Jacaranda Health to test change strategies using PDSA cycles and track their progress toward improvement by reviewing patient exit interview data and plotting these data points on a run chart. All facilities within the QIC met quarterly to share learning from the previous quarter and prepare to test ideas for improvement in new PCMC topics. While QIC teams could work to improve up to eight PCMC topics, all worked on fewer, either because some facilities already showed high performance in a topic (based on patient exit interviews) at the time a topic was introduced in the QIC, or because the facility was unable to effect change in a topic due to structural constraints such as lack of space for a companion to accompany a patient during delivery. Two out of the three facilities focused on two PCMC sub-domains: (1) Dignity and Respect and (2) Communication and Autonomy. The third facility worked on three PCMC sub-domains: (1) Dignity and Respect, (2) Communication and Autonomy, and (3) Supportive Care. A full list of change strategies and associated PCMC sub-domains that were implemented by each health facility is listed in Table 1.

**Table 1.** PCMC topics of improvement and successful change strategies by intervention facility

**Person-centred maternity care topics (PCMC Sub-Domain focus)**	**Change strategies tested**
***Health Facility 1***	
Healthcare providers introduced themselves to clients when they first came to see them (Dignity and Respect)	- Verbal appreciation of staff who introduced themselves to clients - Added topic to staff appraisals - Maternity nurses introduced themselves to three clients as a small scale test to see how clients responded - Nurses spoke to individual peers and told stories about their experiences of introducing themselves to clients to bring others on board - The Medical Superintendent sent a memo to all hospital departments to sensitise them to the need to introduce themselves - Quality Improvement (QI) team members brought other staff cadres on board through peer-to-peer storytelling
Healthcare providers referred to clients by their name (Dignity and Respect)	- Added topic to staff appraisals
Doctors and nurses explained to clients why they were doing examinations or procedures on them (Communication and Autonomy)	- Added topic to staff appraisals
Doctors and nurses explained to clients why they were giving them any medicines (Communication and Autonomy)	- Added topic to staff appraisals
Doctors, nurses and other staff at the facility asked permission/consent before they conducted a procedure on a client (Communication and Autonomy)	- Added topic to staff appraisals
***Health Facility 2***	
Healthcare providers introduced themselves to clients when they first came to see them (Dignity and Respect)	- Staff gave daily reminders to their peers to introduce themselves to clients - A maternity nurse introduced him/herself to clients during the admission process - QI team members sensitised other staff in the facility to the need to introduce themselves to clients
Healthcare providers referred to clients by their name (Dignity and Respect)	- Staff gave daily reminders to their peers to call clients by their name - A maternity nurse called clients by their name during the admission process
Doctors and nurses explained to clients why they were doing examinations or procedures on them (Communication)	- Staff gave daily reminders to their peers that they needed to explain to clients why they were doing examinations and procedures on them - A maternity nurse explained the examinations and procedures a client could expect and why they were needed during the admission process
Doctors and nurses explained to clients why they were giving them any medicines (Communication)	- Staff gave daily reminders to their peers that they needed to explain to clients why they were giving them any medicines - A maternity nurse explained to the client what medicine they were likely to receive and why it was necessary during the admission process
Doctors, nurses and other staff at the facility asked permission/consent before they conducted a procedure on a client (Communication)	- Staff gave daily reminders to their peers that they needed to ask permission/consent before conducting a procedure on a client - A maternity nurse explained to the client what procedures they were likely to need and asked for permission/consent during the admission process
***Health Facility 3***	
Healthcare providers introduced themselves to clients when they first came to see them (Dignity and Respect)	- Maternity nurses on the QI team introduced themselves to admitted patients only. (This was to overcome concerns about a complaint if they had to transfer the client elsewhere for their care)
Healthcare providers referred to clients by their name (Dignity and Respect)	- The topic was introduced at a continuous medical education session
Clients were allowed to have a companion of their choice stay with them during labour (Supportive Care)	- Maternity nurses asked clients if they wanted to have a companion stay with them during labour
Clients were allowed to have a companion of their choice stay with them during delivery (Supportive Care)	- Maternity nurses asked clients if they wanted to have a companion stay with them during labour

### Study participants

Prior to each external round of data collection, an all-female team of trained enumerators piloted survey instruments in participating facilities. Enumerators were stationed across all six facilities and worked closely with facility providers to identify and approach potentially eligible participants. In a private setting within the facility grounds, enumerators introduced the study and obtained written informed consent among interested and eligible participants prior to any study procedures. Eligible criteria included women aged 15–49 who had delivered in the last seven days and had a functional phone of their own (to disburse incentives and allow for follow-up). Participants had the choice to be surveyed at the facility or at home, though no-one opted for a home visit. Cross-sectional data of 491 women (control = 229, intervention = 262) were collected between August and December 2016 to understand baseline PCMC experience of women. The endline was conducted between late October 2018 and early April 2019, during which 677 women were surveyed (control = 352, intervention = 325). In order to evaluate the package of interventions, we assessed baseline and endline means of person-centred care across intervention and control matched facilities. We assumed a 10% change in person-centred care based on current literature, with 80% power for two-sample comparison with two-sided alpha of 0.05. We estimated that this would require a sample of 286 each for the control and intervention arms at baseline and endline.^17^ We attempted to have equal numbers across the baseline and endline phases; however, a doctor’s strike in Kenya in September 2016 slowed recruitment during baseline, thus the slightly lower numbers at baseline compared to endline.

### Study measures

#### Outcome of interest: person-centred maternity care score

PCMC was measured using a PCMC scale, which was developed and validated in Kenya and has demonstrated high content, construct, and criterion validity and good internal consistency and reliability.^15,18^ The 30-item scale covers three PCMC domains: Dignity and Respect (six indicators, with examples including “*did the doctors, nurses, or other healthcare providers call you by your name?*” and “*did the doctors, nurses, and other staff at the facility treat you in a friendly manner?*”); Communication and Autonomy (nine indicators, with examples including “*did the doctors and nurses explain to you why they were giving you any medicine?*” and “*did you feel you could ask the doctors, nurses or other staff at the facility any questions you had?*”); and Supportive Care (15 indicators, including “*do you think there was enough health staff in the facility to care for you?*”). Each indicator can receive a possible score of 3 points (0 “*No, never*”; 1 “*Yes, a few times*”; 2 “*Yes, most of the time*”; 3 “*Yes, all of the time*”). For the full list of PCMC indicators, see Afulani et al.^15^ Responses marked as “*not applicable*” were conservatively recoded to receive the highest possible score. The total PCMC score may range between a minimum of zero and a maximum of 90. The final PCMC score was scaled to a 100-point score.

#### Demographic characteristics and other covariates

We constructed a clinical quality index (possible range from 0 to 22) that captures women’s individual experience of clinical quality. This index combines 22 items that ask each participant whether she received certain procedures or services such as blood pressure, pulse checks, or vaginal examinations during her stay at the facility. Secondary outcomes investigated included how frequently a doctor/nurse visited the participant in the postnatal ward, occurrence of delivery problems, participants’ overall satisfaction with the services received, participants’ quality of care rating, and participants’ intention to use a family planning method in the next six months. We examined socio-demographic factors, pregnancy factors, and facility and provider characteristics that may be associated with PCMC and various outcomes. Socio-demographic factors included age, parity, marital status, age at marriage, age at first pregnancy, number of pregnancies, employment, religion, national wealth quintile, education, and literacy. Pregnancy characteristics such as number of antenatal care visits and problems during pregnancy were also examined. Facility characteristics included facility type, delivery provider gender, type of delivery provider, and the facility’s baseline clinical quality. Baseline clinical quality was captured by the mean clinical quality index score of each facility’s baseline sample.

#### Statistical analysis

We conducted bivariate analyses to compare intervention and control groups pre- and post-intervention on socio-demographic factors, pregnancy factors, facility, and provider characteristics. We evaluated differences across groups by performing cross-tabulations, chi-square tests, and t-tests. To investigate the impact of the intervention on the various outcomes, we conducted a difference-in-differences analysis using models that included main effects of both treatment group and survey round, as well as a two-way interaction term. Ordinary least squares regression was conducted to examine the impact of the QIC on PCMC scores, sub-domains of PCMC, and clinical quality. We tested for homogeneity of variance of residuals using White’s test and used robust standard errors (Eicker-Huber-White) to correct for heteroscedasticity in our models. Logistic regression was employed to assess the effect of the intervention on secondary outcomes. Robust standard errors were used to correct for clustering of participants at the facility level. In addition, all multivariate models were adjusted for age, marital status, parity, employment, education, facility type, type of delivery provider, pregnancy complications, and baseline clinical quality. Analyses were performed using Stata SE 15.1 and an alpha level of 0.05 was established for statistical significance.

#### Data triangulation: stakeholder feedback

Twelve months after the completion of the QIC, the study team triangulated study results by holding dissemination meetings with QI teams and/or relevant staff at both intervention and control facilities. Study researchers facilitated a group discussion and took detailed notes with 4–11 facility staff in attendance per meeting. The meetings sought to share findings with participating facilities, contextualise the findings, and give facilities an opportunity to provide feedback on the results and/or how to improve future QI endeavours.

### Ethical approval

All activities under this study were approved by the Institutional Review Board of the University of California, San Francisco (#15-18008, January 29, 2016) and the Kenya Ethics Medical Review Institute (#Non-KEMRI 526, May 25, 2016). The study received all necessary national and county- and facility-level approvals, including from the National Commission for Science, Technology and Innovation (NACOSTI, July 1, 2016).

## Results

### Socio-demographic characteristics and PCMC scores

Socio-demographic characteristics, facility characteristics, and clinical quality were assessed at baseline and differences across control and intervention facilities are presented in Table 2.

**Table 2.** Characteristics of participants, by survey round

	Baseline				Endline			
	Control	Intervention	Total	P-value	Control	Intervention	Total	P-value
**Total number in group**	229	262	491		352	325	677	
**Age**								
Mean	25.94	25.23	25.56	0.108	26.58	25.93	26.27	0.106
(SD)	(4.54)	(5.1)	(4.85)		(5.02)	(5.47)	(5.25)	
**Parity**								
Mean	2.23	2.11	2.16	0.252	2.32	2.25	2.29	0.488
(SD)	(1.12)	(1.19)	(1.16)		(1.16)	(1.52)	(1.34)	
**Marital Status**								
Single (%)	11.8%	12.6%	12.2%	0.208	14.8%	13.2%	14.0%	0.529
Cohabiting/Partnered (%)	17.0%	11.8%	14.3%		0.6%	1.2%	0.9%	
Married (%)	70.3%	73.7%	72.1%		84.1%	84.0%	84.0%	
Widowed (%)	0.4%	0.0%	0.2%		0.3%	0.3%	0.3%	
Divorced/Separated (%)	0.4%	1.9%	1.2%		0.3%	1.2%	0.7%	
**Occupation**								
Casual Labour (%)	14.8%	18.3%	16.7%	0.252	8.2%	8.3%	8.3%	0.706
Salaried Worker (%)	9.6%	10.3%	10.0%		14.8%	16.6%	15.7%	
Self-employed in petty trade (%)	27.1%	18.7%	22.6%		27.8%	27.7%	27.8%	
Self-employed small scale industry (%)	2.2%	3.1%	2.6%		2.8%	2.2%	2.5%	
Unemployed/homemaker (%)	46.3%	49.6%	48.1%		46.3%	44.6%	45.5%	
Missing (%)	0.0%	0.0%	0.0%		0.0%	0.6%	0.3%	
**Religion**								
None (%)	0.0%	0.8%	0.4%	0.317	0.3%	0.6%	0.4%	0.203
Catholic (%)	23.6%	27.1%	25.5%		25.9%	28.0%	26.9%	
Protestant/Pentecostal (%)	60.3%	58.0%	59.1%		52.0%	53.2%	52.6%	
Other Christian (%)	15.7%	12.6%	14.1%		19.6%	17.8%	18.8%	
Muslim/other religion (%)	0.4%	1.5%	1.0%		2.3%	0.3%	1.3%	
**Highest grade/class completed**								
No school/Primary (%)	42.4%	35.5%	38.7%	0.289	35.8%	32.0%	34.0%	0.464
Post-primary/vocational/Secondary (%)	41.9%	47.7%	45.0%		44.6%	45.2%	44.9%	
College or above (%)	15.7%	16.8%	16.3%		19.6%	22.8%	21.1%	
**Number of ANC visits**								
Less than 4 (%)	41.5%	40.5%	40.9%	0.247	41.2%	37.2%	39.3%	0.058
4 or 5 (%)	50.2%	46.6%	48.3%		48.3%	45.5%	47.0%	
6 plus (%)	7.4%	11.8%	9.8%		10.2%	16.3%	13.1%	
Missing (%)	0.9%	1.1%	1.0%		0.3%	0.9%	0.6%	
**Problems during pregnancy**								
No (%)	85.6%	82.4%	83.9%	0.344	75.6%	62.8%	69.4%	0.000
Yes (%)	14.4%	17.6%	16.1%		24.4%	37.2%	30.6%	
**Problems during delivery**								
No (%)	91.7%	81.3%	86.2%	0.001	89.5%	84.9%	87.3%	0.075
Yes (%)	8.3%	18.7%	13.8%		10.5%	15.1%	12.7%	
**Problems after delivery**								
No (%)	88.2%	88.5%	88.4%	0.907	86.4%	79.7%	83.2%	0.020
Yes (%)	11.8%	11.5%	11.6%		13.6%	20.3%	16.8%	
**Facility Type**								
Government hospital	100.0%	69.1%	83.5%	0.000	100.0%	63.4%	82.4%	0.000
Gov't Health Center (%)	0.0%	30.9%	16.5%		0.0%	36.6%	17.6%	
**Delivery Assistant**								
Nurse/Midwife (%)	64.2%	36.3%	49.3%	0.000	47.7%	39.1%	43.6%	0.054
Doctor/Clinical Officer (%)	19.2%	38.5%	29.5%		34.9%	36.0%	35.5%	
More than one skilled assistant (%)	15.7%	24.8%	20.6%		15.6%	22.2%	18.8%	
Other/Non-skilled attendant (%)	0.9%	0.4%	0.6%		1.7%	2.8%	2.2%	
**Gender of delivery assistant**								
Male (%)	12.2%	17.6%	15.1%	0.076	25.3%	24.9%	25.1%	0.001
Female (%)	72.5%	63.0%	67.4%		69.0%	60.9%	65.1%	
Both (%)	15.3%	19.5%	17.5%		5.7%	14.2%	9.7%	
**Clinical Quality of Care Index**								
Mean	12.68	14.06	13.42	0.001	13.76	13.22	13.5	0.164
SD	(4.4)	(4.8)	(4.7)		(4.9)	(5.0)	(5.0)	

At baseline, the intervention group average total PCMC score was slightly lower than controls (Table 3). Of the sub-domains, only Dignity and Respect displayed a higher mean score for the intervention group compared to controls at baseline. Between baseline and endline, the mean total PCMC score decreased for the intervention group, yet increased for the control group. Similar trends over time were observed for each of the sub-domains.

**Table 3.** Mean PCMC scores, by survey round and sub-domain of PCMC

	Baseline				Endline			
	Control (N=229)		Intervention (N=262)		Control (N=352)		Intervention (N=325)	
	Mean	SD	Mean	SD	Mean	SD	Mean	SD
**Total PCMC score**								
PCMC total sum (30-indicator final scale)	67.14	(12.75)	65.68	(14.71)	71.4	(17.34)	63.98	(16.99)
Dignity and Respect domain subtotal	78.68	(15.97)	80.85	(17.01)	82.62	(20.50)	75.71	(19.35)
Communication and Autonomy domain subtotal	54.34	(16.44)	56.79	(18.49)	61.84	(22.90)	56.13	(20.85)
Supportive Care domain subtotal	70.2	(13.75)	64.95	(16.17)	72.65	(16.92)	64.01	(18.48)

**Means are re-scaled from 0-100 range to assist with interpretability

### Impact of the intervention

**Figure 2.**
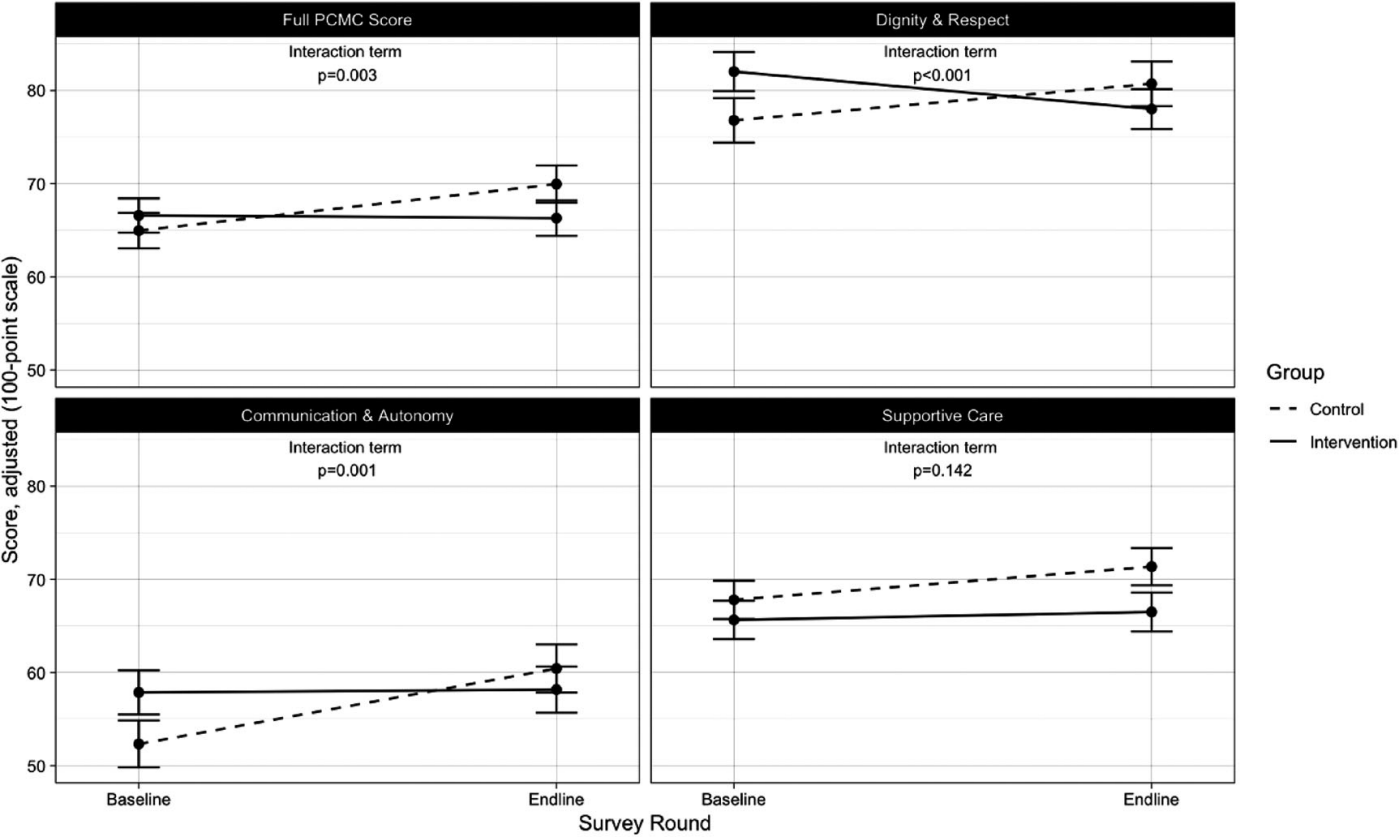
Impact of intervention on PCMC scores *Adjusted for age, marital status, total number of births, employment, education, wealth quintile, facility type, provider type, pregnancy complications, and baseline clinical quality

Between baseline and endline and compared to control facilities, participants at intervention facilities reported significantly lower total PCMC and subdomain scores for all except the Supportive Care domain (Figure 2).

Across time, intervention facilities’ average total PCMC score decreased 5.3 points (95% CI: −8.8, −1.9) relative to control facilities, adjusting for socio-demographic, and facility characteristics (Table 4). Women who were attended by a doctor/clinical officer or more than one skilled assistant reported lower PCMC scores than those attended to by a nurse/midwife. Pregnancy complications and baseline clinical quality were also associated with lower and higher PCMC scores, respectively.

**Table 4.** Difference-in-difference analyses to assess the impact of the intervention on PCMC scores, by sub-domains of person-centered maternity care^a^

	Full PCMC score						Dignity & Respect			Communication & Autonomy domain			Supportive Care domain		
	Unadjusted			Adjusted*			Adjusted*			Adjusted*			Adjusted*		
	**Cofficient**	**95% CI**	**p-value**	**Cofficient**	**95% CI**	**p-value**	**Cofficient**	**95% CI**	**p-value**	**Cofficient**	**95% CI**	**p-value**	**Cofficient**	**95% CI**	**p-value**
Survey Round															
Baseline	0	(ref)		0	(ref)		0	(ref)		0	(ref)		0	(ref)	
Endline	4.26	(1.62, 6.90)	0.002	4.91	(2.40, 7.43)	0.000	3.91	(0.84, 6.98)	0.013	7.98	(4.69, 11.27)	0.000	3.48	(0.90, 6.05)	0.008
Intervention assignment															
Control	0	(ref)		0	(ref)		0	(ref)		0	(ref)		0	(ref)	
Intervention	-1.45	(-4.27, 1.36)	0.311	1.54	(-1.34, 4.42)	0.294	5.20	(1.81, 8.59)	0.003	5.42	(1.67, 9.17)	0.005	-2.25	(-5.45, 0.94)	0.167
Endline-Intervention Interaction Term	-5.96	(-9.66, -2.27)	0.002	-5.33	(-8.76, -1.90)	0.003	-7.91	(-12.02, -3.80)	0.000	-7.93	(-12.40, -3.46)	0.001	-2.74	(-6.35, 0.87)	0.137

Over time, the intervention was significantly associated with a 1.8-point decrease in clinical quality index (95% CI: −2.9, −0.7) (Table 5). The intervention was also associated with a 73% decreased odds of frequent provider visits (95% CI: 83%, 56%) over time. Women in intervention facilities were 55% less likely to plan on using family planning (95% CI: 79%, 4%) relative to controls across time. There were no significant differences in changes over time between intervention and control facilities with respect to delivery problems, quality of care, and overall satisfaction with care.

**Table 5.** Impact of QI intervention on other health outcomes^a^

	Survey Round	Intervention	Interaction term
**Clinical Quality Index**			
Coefficient (OLS)	1.20	1.78	-1.80
95%CI	(0.42, 1.98)	(0.95, 2.61)	(-2.89, -0.69)
p-value	0.003	0.000	0.002
**Frequency of provider visits (3 or more per day)**			
aOR	1.94	2.55	0.27
95%CI	(1.36, 2.76)	(1.72, 3.78)	(0.17, 0.44)
p-value	0.000	0.000	0.000
**Quality of care rating**			
aOR	0.67	0.67	2.00
95%CI	(0.29, 1.55)	(0.28, 1.60)	(0.73, 5.51)
p-value	0.349	0.364	0.180
**Delivery Problems**			
aOR	1.02	1.93	0.55
95%CI	(0.56, 1.86)	(1.07, 3.49)	(0.26, 1.17)
p-value	0.947	0.029	0.121
**Overall satisfaction with care**			
aOR	0.76	0.98	1.19
95%CI	(0.35, 1.66)	(0.43, 2.31)	(0.45, 3.15)
p-value	0.494	0.995	0.722
**Planning to use Family Planning**			
aOR	1.26	1.18	0.45
95%CI	(0.73, 2.17)	(0.64, 2.16)	(0.21, 0.96)
p-value	0.411	0.603	0.039

a. All estimates were adjusted for age, marital status, total number of births, employment, education, facility type, delivery provider, pregnancy complications, and baseline clinical quality of care. Robust standard errors were used.

### Contextual information during the intervention

The QIC itself continued for much longer than is typical due to interruptions associated with two national strikes of doctors and nurses. The QI activity within facilities during these setbacks, including when to break and resume during strikes, was guided by the team members who worked within the health facilities. Over this timeframe, some emergent QI champions moved to different departments and/or facilities, interrupting the intervention’s momentum and potentially reducing enthusiasm among remaining active QI team members, as well as facility and county leadership. In addition, there was a notable focus by the Kenyan national and county governments to improve respectful maternity care, particularly in Nairobi County, where two of the three control facilities were located, compared to only one of three intervention facilities. These two facilities also underwent county-led upgrades to infrastructure such as improved equipment and supplies, and an increased bed capacity. One of the Nairobi control facilities also faced a change in management, as well as increased staffing, specifically in the maternity department starting in 2017. The remaining control facility in Kiambu was identified to also participate in county-led trainings focused on improving both respectful maternity care and clinical quality of maternity care. The county also provided additional staff, such as nurses and clinical officers, who were involved with provision of maternity during the study period.

Leadership engagement within a facility and continuity of QI team members were key factors in the success of the QIC intervention. Facilities that had less leadership buy-in were marked by low weekly QI meeting attendance and low adherence to assigned activities by QI team members in carrying out PDSA cycles and collecting internal data. Health facilities that had changes in the QI team membership due to reshuffling of departmental staff or relocation of staff to other facilities struggled to maintain the vision of the QI work within the team and to get buy-in from other staff in the departments whose participation was crucial to scaling up tests of ideas to improve PCMC topics.

## Discussion

This study found that: (1) the QIC was not successful in improving PCMC scores in public health facilities in two urban/peri-urban counties in Kenya; and (2) the intervention was associated with a decline in other outcomes including clinical quality scores, odds of frequent provider visits, and women reporting planning to use a family planning method in the future; moreover, no differences were detected with respect to delivery problems, perceived quality of care or overall satisfaction with care across intervention and control facilities over time.

Our findings contribute to literature on improving women’s experiences of care by examining the impact of a QI intervention in a low-resource setting. Our negative findings align with other research finding mixed results of PCMC interventions.^8^ Associated implementation challenges of QICs and PCMC may have produced negative results. Specifically, the focus on patient experience only as opposed to patient experience combined with clinical trainings may have contributed to negative results. Recent studies show that provider trainings emphasising learning, practising, and reflection of respectful maternity care (RMC) in the context of stressful emergency obstetric simulations have the potential to improve person-centred care.^19^ That is, the emphasis of both clinical training in tandem with training in person-centred care may be needed. Future studies may want to coordinate QICs that include both a provider clinical training and person-centred care model.

### Clinical implications

The dissemination meetings with facilities post-intervention confirmed that our assumptions related to negative findings were contextually sound. Overall, all intervention facilities found results surprising because of the general sense that the QI approach was helpful; however, they also offered a number of potential explanations as to why the intervention may not have been successful.

The PCMC sub-domains of focus for the QI intervention itself may have been problematic given the health systems in which the interventions were implemented. First, the most common explanation for the lack of intervention impact was related to health system constraints, such as lack of resources from the government (i.e. inconsistent supply of essential medicines, etc.) and insufficient staff to patient ratio. For example, behaviours that could lead to PCMC improvements within the subdomain of Dignity and Respect, such as staff introductions or learning a patient’s name, may not have been viewed as a priority despite the ease with which these approaches may be implemented, particularly when clinical staff were navigating challenges related to staff shortages coupled with unmanageably high patient volumes. Certain PCMC behaviours related to the subdomain of Communication and Autonomy, such as explaining clinical procedures and tests, or why medicines were being administered, may have been additionally difficult to integrate into care approaches given significantly high patient-to-staff ratios and competing clinical priorities to ensure the physical health and well-being of the mother or child. Interestingly, we note that baseline and endline results remain fairly static in the subdomain of Supportive Care; with interventions focused on allowing a companion to be present during labour and delivery. It is possible that this result was influenced specifically by a better balanced patient-to-staff ratio, more physical space, and lower delivery volumes within the smallest intervention facility (Health facility 3) Results from in-depth interviews conducted with 32 QI team members following the QI collaborative have been published previously and found that many respondents discussed health system constraints in taking a PCMC approach.^20^ Challenges to delivering high-quality person-centred care included disproportionate staff-to-patient ratios, high staff turnover, and lack of space. Future work aimed at securing improvements in PCMC within existing resources needs to consider health system challenges and minimum standards of available resources required for improvement to be theoretically possible.

Second, a few staff believed providers may have found it challenging to embrace the new concept of PCMC as critical to their responsibilities, and that the time perceived to be taken to implement these activities could impede their ability to carry out clinical duties successfully and efficiently. Providers may be more willing or able to prioritise interventions that are designed to improve their ability to provide high-quality clinical care specifically, or address infrastructure challenges and inefficiencies within their control. Indeed, qualitative in-depth interviews conducted at baseline with healthcare providers have been published previously.^21^ Results found that healthcare providers rationalised abuse (e.g. physical force or verbal abuse) by indicating that these behaviours are required to save the life of a mother and their new-born. Healthcare providers described ensuring that the safety of the mother and their new-born is their main responsibility, even if that occurs in the context of poor person-centred care. Future QI efforts should design a QIC that meet the needs of both patients and providers. In particular, future efforts need to train providers on PCMC in addition to clinical quality care and, importantly, how they can be delivered in tandem despite health system constraints.

Third, the QI approach may have been overly cumbersome to implement. Almost all staff discussed how the requirement to obtain weekly feedback from patients on aspects of their PCC experiences through exit interviews was time-intensive – both for the QI team and for women who had just delivered. These circumstances may have contributed to burnout and declining participation by QI team members. Attendance at QI team meetings was reported to be challenging for members with key clinical roles due to poor staff-to-patient ratios. Moreover, the two national strikes of doctors and nurses added further strain on the providers who worked during the strike period, with varying degrees of impact on healthcare delivery and thus QIC work. During each strike, the staff turnover and subsequent movement of the emergent QI champions to different departments and/or facilities meant that any incoming providers were new to the QI intervention, may not have received adequate on-boarding, and may not have prioritised such activities. In-depth qualitative interviews published previously corroborate these explanations as team members described health system constraints such as high turnover that led to less continuity of staffing in the QI collaborative and challenges with time constraints on attending QI meetings. Interestingly, in spite of these challenges, QIC team members still highlighted that participation in the QIC facilitated development of important perceived benefits such as improved interpersonal communication, greater satisfaction with their work, and enhanced understanding of patient preferences.^20^

Concurrent QI activities, particularly at control facilities, may also explain our results. Across both control and intervention facilities, staff reported broader QI interventions, including effective communication and respectful maternity care, occurring over the duration of the project from a variety of stakeholders, including government. It is notable that government-led QI initiatives had a strong buy-in by the facilities. Therefore, while the intervention facilities may have struggled with implementation challenges related to the QIC, at least two of three control facilities received significant support from the government for infrastructure and equipment during the intervention period. These improvements may have contributed to differential improved clinical quality.

### Research implications

While QICs are often cited as viable approaches to improve health outcomes and processes in resource-constrained settings because they focus on working within the means and context of a health system, these approaches may not be appropriate to improve PCMC behaviours among clinical staff in all contexts. This study highlights important potential lessons learned and offers directions for future research. First, should future studies choose to implement a QIC model, they should adapt the QI approach undertaken in this study to address facility buy-in, time and resource constraints, the burden of data collection for PCMC topics, as well as potential trainings in clinical quality, to assess whether more positive findings might be reported. Because women reported higher levels of person-centred care with midwives as opposed to doctors, specific trainings aimed towards doctors to provide respectful care may be needed. Second, process evaluations would be critical to examining specific facilities and external factors essential to the success of the QI approach. Topics may include facility buy-in, challenges in existing infrastructure and clinical care, as well as external factors such as national strikes or government/NGO support.

### Strengths and limitations

This study is, to our knowledge, the first to implement a QIC to improve PCMC. Strengths of the study are that it includes pre- and post-intervention data for both control and intervention facilities. Additionally, it includes rich data on both clinical and experiential quality of care using a validated measure of PCMC. We were also able to triangulate data by conducting dissemination meetings with participating facilities.

Limitations to the study include it being carried out in only six facilities in two counties in Kenya, Nairobi, and Kiambu, both of which are urban/peri-urban. The research was undertaken over a 33-month period when the health service experienced considerable political turbulence and differential investment. However, disruptions in service and broader political factors are reflective of real-world events that are likely to play a role in other studies involving QI efforts. These findings do not highlight how a QIC might perform in rural areas, in other regions of Kenya, or over a shorter period of time. Moreover, it should be noted that the three healthcare facilities included in the intervention were motivated to improve patient experience and, most importantly, had facility leadership and management that oversaw the development and continuation of a QIC. Related, we included facilities that had support from local county officials to participate in the intervention. Facility selection also included facilities that were somewhat higher volume (i.e. average of at least 50 deliveries per month). Therefore, the generalisability of these findings are limited. Healthcare facilities with lower resources, lower volume of deliveries, and without adequate leadership to implement monitoring activities and a QIC may find it difficult to implement this type of intervention. On the other hand, it is also possible that facilities with lower volume of deliveries may have more capacity to implement QIC activities if there are better patient to provider ratios and more time to implement strategies. Differences in these types of factors might be a possible explanation for the lack of effect of the intervention. Lastly, while we reached adequate sample size for the endline sample for intervention and control groups, we had slightly lower samples than required for baseline groups based on sample size calculations due to a strike. Therefore, larger samples are needed in future intervention studies to detect differences in PCMC of 10% or lower.

## Conclusions

While improving the quality of women’s experiences during childbirth is a critical component to ensure comprehensive, high-quality maternity care experiences, and outcomes, this study highlights the complexity of improving PCMC. There is a need for further research to identify which intervention methods will appropriately and effectively improve PCMC in resource-constrained public facility settings. Despite these negative results, it remains a public health priority that women’s experiences of delivery care improve globally and that all women have access to respectful and dignified care.

## Data Availability

The datasets used and/or analysed during the current study are available from the corresponding author on reasonable request.
